# Salvianolate ameliorates oxidative stress and podocyte injury through modulation of NOX4 activity in db/db mice

**DOI:** 10.1111/jcmm.16165

**Published:** 2020-12-17

**Authors:** Yiran Liang, Hong Liu, Yi Fang, Pan Lin, Zhihui Lu, Pan Zhang, Xiaoyan Jiao, Jie Teng, Xiaoqiang Ding, Yan Dai

**Affiliations:** ^1^ Department of Nephrology Zhongshan Hospital Fudan University Shanghai China; ^2^ Shanghai Institute of Kidney and Dialysis Shanghai China; ^3^ Shanghai Key Laboratory of Kidney and Blood Purification Shanghai China

**Keywords:** AMPK, diabetes nephropathy, mitochondria, NADPH oxidases 4, podocyte injury, reactive oxygen species, Salvianolate

## Abstract

Podocyte injury is associated with albuminuria and the progression of diabetic nephropathy (DN). NADPH oxidase 4 (NOX4) is the main source of reactive oxygen species (ROS) in the kidney and NOX4 is up‐regulated in podocytes in response to high glucose. In the present study, the effects of Salvianolate on DN and its underlying mechanisms were investigated in diabetic db/db mice and human podocytes. We confirmed that the Salvianolate administration exhibited similar beneficial effects as the NOX1/NOX4 inhibitor GKT137831 treated diabetic mice, as reflected by attenuated albuminuria, reduced podocyte loss and mesangial matrix accumulation. We further observed that Salvianolate attenuated the increase of Nox4 protein, NOX4‐based NADPH oxidase activity and restored podocyte loss in the diabetic kidney. In human podocytes, NOX4 was predominantly localized to mitochondria and Sal B treatment blocked HG‐induced mitochondrial NOX4 derived superoxide generation and thereby ameliorating podocyte apoptosis, which can be abrogated by AMPK knockdown. Therefore, our results suggest that Sal B possesses the reno‐protective capabilities in part through AMPK‐mediated control of NOX4 expression. Taken together, our results identify that Salvianolate could prevent glucose‐induced oxidative podocyte injury through modulation of NOX4 activity in DN and have a novel therapeutic potential for DN.

## 1 INTRODUCTION

Diabetic nephropathy (DN) is the leading cause of the end‐stage renal disease (ESRD), accounting for a high burden of morbidity and mortality.^1^ Podocytes are terminally differentiated cells and podocyte injury, as a result of cell detachment and apoptosis, has been described to be an early event in DN.^2^, ^3^ There is abundant evidence suggesting that oxidant injury to podocytes may play a pivotal role in the pathogenesis of DN. Although multiple pathways may result in ROS generation, the NADPH oxidases (NOXs) appear to be the most important contributor to mediate glomerular injury in diabetes.^4^ It has been documented that NOXs, particularly the isoform NOX4 is a pathologically relevant source of ROS in glucose‐induced podocytes with an associated increase in ROS and albuminuria.^5^, ^6^ In particular, mitochondrial Nox4 from podocytes might be a key inducer of mitochondrial dysfunction and podocyte apoptosis in DN.^7^, ^8^ Therefore, novel approaches to block NOX4‐derived ROS in the podocytes may hold the promise to rescue the kidney from oxidative damage and prevent subsequent progression of DN.

Salvianolate (SAL) is a prescribed Chinese medicine derived from Danshen (the root of *Salvia miltiorrhiza*) and widely used in the clinic in treating cardiovascular diseases.^9^, ^10^, ^11^ As the major bioactive components (content >85%) of SAL, the pharmacological actions of Salvianolic acid B (Sal B) have been extensively investigated.^12^, ^13^, ^14^ Recently, Sal B has also been proved to ameliorate hyperglycaemia and dyslipidemia in db/db mice through therapeutic modulation of AMP‐activated protein kinase (AMPK) pathway.^10^ AMPK is activated by phosphorylation of a critical threonine residue (Thr^172^) in the catalytic α‐subunit upon alterations of the cellular AMP/ATP ratio.^15^ AMPK activation may protect albuminuria and podocyte permeability via inhibition of NADPH oxidase activation and the expression of NOX subunits, including NOX4.^16^, ^17^, ^18^ Substantial evidence suggests that SAL could improve the activity of antioxidant enzymes due to its polyphenolic structure (Figure S1). Our team has demonstrated previously that Sal B exerted anti‐oxidative effects to protect renal tubular cells from iodinated contrast media‐induced acute kidney injury (AKI).^19^ Based on these clues, it is reasonable to hypothesize that SAL inhibited high glucose‐induced NOX4‐based ROS generation in podocytes through AMPK‐mediated control of Nox4 expression, thereby ameliorating podocyte injury in DN. However, there are no reports on the role of SAL against podocyte injury and progression of DN.

Based on these findings, here we attempt to address (a) The therapeutic effects of SAL in db/db diabetic mice; (b) the role of Sal B on NOX4 activity, NOX4‐derived mitochondrial ROS production and apoptosis of podocytes under high glucose; and (c) whether AMPK is required for Sal B to attenuate NOX4‐induced podocyte injury in DN. Key findings in vivo studies were confirmed in vitro using human podocytes.

## 2 MATERIALS AND METHODS

### 2.1 Reagents

Salvianolate injection (Lot. number 16110121) was provided by Shanghai Green Valley Pharmaceutical Co., Ltd. It contains Salvianolic acid B (Sal B, ≥85%) and its analogs, rosmarinic acid (RA ≥ 10.1%) and lithospermic acid (LA, ≥1.9%), as active components. Salvianolic acid B (purity >98%) was purchased from the Shanghai Oriental Pharmaceutical Technology Industrial Co., Ltd. and dissolved in saline. NOX1/4 dual inhibitor GKT137831 (purity >99.43%) was purchased from MedChemExpress Co., Ltd. The Glutathione (GSH) and malondialdehyde (MDA) kits were purchased from Nanjing Jiancheng Bioengineering Institute (Nanjing, China). The other chemicals used were of reagent grade from Sinopharm Chemical Reagent Co., Ltd.

### 2.2 Animal studies

Eight‐week‐old male diabetic *db/db* mice (C57BLKS/J‐leprdb/leprdb) and their lean littermate control *db/m* were purchased from the Shanghai Research Center for Model Organisms (Shanghai, China). Mice were then divided randomly into six groups (n = 6 in each group): (a) non‐diabetic control *db/m* mice, (b) control *db/db* mice treated with vehicle, (c) *db/db* mice treated with SAL, (d) *db/m* mice treated with SAL, (e) *db/db* mice treated with GKT137831, (f) *db/db* mice treated with corn oil. After being dissolved with 0.9% saline, SAL was injected intraperitoneally (80 mg/kg three times a week) to the mice and continued for 16 weeks. Mice are treated with 60 mg/kg of GKT137831 dissolved in corn oil by oral gavage (three times a week). SAL dosage used in our experiment was chosen according to previous animal studies and our earlier pilot study.^20^, ^21^ For all experiments, glycaemia and body weight were monitored in diabetic animals twice per week. Individual mice were placed in a metabolic cage to collect 24 hours urine samples. After the mice were killed at 24 weeks of age, blood serum, kidney tissues were collected for subsequent analyses. Iron beads were perfused in one kidney for glomerular isolation; the other kidney was perfused with 4% paraformaldehyde for histology and immunostaining. All animal studies were performed according to the protocols approved by the Institutional Animal Care and Use Committee of Fudan University and conducted following the UK Animals (Scientific Procedures) Act, 1986 and associated guidelines.

### 2.3 Western blot analysis

The kidneys and cells were lysed with RIPA solution containing 1% NP40, 0.1% SDS, 100 mg/mL PMSF, Complete Protease Inhibitor Cocktail Tablets (Roche) on ice. Proteins were detected using specific antibodies as following: p‐AMPKα^Thr172^ (#50081, 1:1000), total AMPKα (#5831, 1:1000), β‐actin (#3700, 1:5000) were from Cell Signaling Technology; NOX4 (ab133303, 1:1000) was from Abcam. Densitometry analysis for the quantification of Western blots was performed as described previously.^22^

### 2.4 Real‐time PCR

Quantitative reverse‐transcriptase PCR was performed using SYBR Green Master Mix (Premix Ex TaqTM TaKaRa and the Applied Biosystems 7500 system). PCR primers were designed using Primer‐Blast (NCBI) to span at least one intron of the targeted gene; the sequences of these primers are listed in Table S1. *C*_t_ values of target genes were normalized to β‐actin and presented as fold increase compared to the reference experimental group using the $2^{‐\Delta \Delta C_{T}}$ method.

### 2.5 Kidney histology

Kidney samples were fixed in 10% formalin and embedded in paraffin. The glomerular volume and mesangial area were determined by examining periodic acid‐Schiff‐stained sections. The relative mesangial area was expressed as the mesangial‐to‐glomerular surface area (percentage). Twenty glomeruli per mice and six mice from each group were counted. For transmission electron microscopy (TEM), kidney cortex samples fixed in 2.5% glutaraldehyde were divided into sections, mounted on a copper grid, and then the images were acquired using a Hitachi H7650 microscope (Hitachi). Quantification of foot process (FP) effacement and glomerular basement membrane (GBM) width was performed using ImageJ software on digitized TEM images, as previously described.^22^

### 2.6 Urine albumin and creatinine analysis

Urine albumin was measured using an ELISA kit (Bethyl Laboratory Inc). Urine creatinine levels were measured in the same samples using a colorimetric assay kit (Bioassays Systems). The urine albumin excretion rate is expressed as the ratio of albumin to creatinine.

### 2.7 Glomerular isolation

Mice glomeruli were isolated as previously reported by perfusion of iron oxide.^22^ The purity of the glomeruli isolate was verified under light microscopy and by Western blot for podocyte‐specific markers, as described elsewhere.

### 2.8 Human Podocyte Cell Line

A conditionally immortalized human podocyte cell line (courtesy of Dr Moin Saleem) was cultured as previously described.^23^

### 2.9 Measurement of intracellular and mitochondrial ROS level

To investigate intracellular ROS production, differentiated podocytes cultured under normal glucose (glucose 5.5 mM, supplemented with 29.5 mM mannitol as high osmolarity control) and high glucose (35 mM) were treated with Sal B or vehicle for 24 hours. Cells were then washed with PBS and incubated with 10 μM of DCFH‐DA (Thermo Fisher) for 30 minutes at 37°C. ROS generation was determined by fluorescence intensity with the setting of 502 and 530 nm for excitation and emission wavelengths, respectively. To investigate mitochondrial‐derived ROS, cells were incubated with 5 µm MitoSOX™ reagent working solution for 10 minutes at 37°C, protected from light. MitoSOX fluorescence was measured at 510 nm excitation and 588 nm emissions using a confocal microscope (Zeiss LSM 700).

### 2.10 Assessment of NADPH oxidase activity and oxidative stress‐related enzymes

NADPH oxidase activity was assessed using a lucigenin chemiluminescence assay as previously described.^24^ Serum‐deprived podocytes and glomeruli isolated from the kidney cortex were washed by ice‐cold HBSS lysis buffer containing protease inhibitor cocktail. Cell suspensions or renal lysates were homogenized with 100 strikes in iced Krebs buffer. The lucigenin‐derived chemiluminescence assay was used to determine NAPDH activity in total protein homogenates. To start the assay, 20 µg of protein homogenates was transferred to each well followed by adding 5 µM Lucigenin (Santa Cruz), 100 µM NADPH (Cayman Chemical). After 10 minutes at 37°C for dark adaptation, the light emission was continuously monitored using a luminometer. The NADPH activity was expressed as relative light units per milligram protein. The activities of MDA, GSH were examined by spectrophotometry using respective detection kits (Jiancheng).

### 2.11 Podocytes with siRNA‐mediated knockdown of AMPKα and NOX4

To knock down AMPKα expression, conditionally immortalized human podocytes cultured at 37°C were infected with 100 nM siRNA‐AMPKα or 100 nM siRNA‐NOX4 using Lipofectamine 3000 for 48 hours to eliminate AMPKα or NOX4 expression, and cells transfected with siRNA‐NC were set as the control (Ruibo Biotechnology).

### 2.12 Immunostaining

Immunostaining was performed on 4‐μm paraffinized sections as previously described.^25^ The samples were incubated with primary antibodies at 4°C overnight and then with biotinylated second antibodies, followed by incubation with an avidin‐biotin‐peroxidase complex; they were developed using substrate provided by Vector Laboratories. The following antibodies were used in this study: anti‐mouse WT‐1 (ab220212, 1:50) and anti‐mouse 8‐OHdG (ab48508, 1:100). For quantification of WT‐1‐positive and 8‐OHdG‐positive nuclei, 15 glomeruli per mice and four mice from each group were examined to calculate the number of stained nuclei.

### 2.13 TUNEL assay

DeadEnd Colorimetric TUNEL System from Promega was used to detect apoptotic cells on formalin‐fixed, paraffin‐embedded kidney sections. The manufacturer's protocol was used to processes the sections. The number of TUNEL‐positive cells from 20 areas of random glomeruli was counted under a light microscope.

### 2.14 Flow cytometry assay of apoptosis

The ratio of podocyte apoptosis was determined by flow cytometric analysis using an Annexin V‐FITC/propidium iodide (PI) kit (BD Bioscience) according to the manufacturer's instructions. All flow cytometry data were acquired on an ACEA NovoCyte (ACEA Biosciences) and analysed with the NovoExpress software.

### 2.15 Immunofluorescence confocal microscopy

Mitochondrial morphology was examined in podocytes stained with 100 nM Mitotracker deep red probe (ThermoFisher). After a wash with PBS, podocytes were fixed in 4% formaldehyde. Then cells were incubated with primary anti‐human NOX4 antibody (ab133303) at 4°C overnight followed by incubated with Alexa Fluor 488 labelled secondary antibody (1:200, Invitrogen) for 2 hours at room temperature. Hoechst was used for nuclei staining. Images were observed and recorded on a Zeiss LSM 700 confocal microscope (Germany).

### 2.16 Statistical Analysis

Data are expressed as mean ± SEM. The unpaired ANOVA was used when comparing between groups for treatment conditions using the GraphPad Prism software. *P*‐value < .05 was considered statistically significant.

## 3 RESULTS

### 3.1 Effects of SAL on the biochemical parameters of diabetic mice

To determine the effects of SAL in DN, SAL (80 mg/kg three times a week) or saline was administrated to db/db diabetic mice or non‐diabetic db/m mice for 16 weeks. As shown in Table 1, we summarized the body weight, kidney weight, fasting blood glucose level and albumin level of db/m and db/db mice treated with either saline or SAL injection. After 16 weeks of treatment, db/db mice treated with saline developed almost 7 times higher albuminuria level (db/db vs db/m: 212.30 ± 4.5 vs 32.2 ± 1.2, *P* < .05) and 3.5 times higher blood glucose level (db/db vs db/m: 28.20 ± 3.00 vs 8.15 ± 1.00, *P* < .05) than non‐diabetiic db/m mice. No significant difference in body or kidney weight was observed between SAL and saline treated db/db mice. Compared to saline treated db/db mice, urinary albumin excretion as assessed by urinary albumin to creatinine ratio in SAL treated db/db mice was significantly lower (SAL treated db/db vs saline treated db/db: 54.9 ± 1.9 vs 212.30 ± 4.5, *P* < .001), whereas the reduction of blood glucose in SAL treated db/db mice did not show a significant difference.

**TABLE 1:** Biochemical parameters of diabetic mice

Parameter	db/m + vehicle	db/m + SAL	db/db + vehicle	db/db + SAL
Fasting blood glucose (mM)	8.15 ± 1.00	7.90 ± 3.20	28.20 ± 3.00^a^	20.50 ± 2.80^a^
Body weight (BW, g)	26.74 ± 1.20	27.32 ± 2.01	54.21 ± 3.24^a^	52.90 ± 2.83^a^
KW/BW (mg/g)	11.42 ± 1.98	10.32 ± 1.54	9.4 ± 1.03^a^	9.8 ± 1.56^a^
Serum creatinine (mg/dL)	42.25 ± 1.93	51.50 ± 1.32	58.25 ± 1.93^a^	54.50 ± 1.71^a^
Urine albumin to creatinine (mg/g)	32.2 ± 1.2	30.8 ± 1.4	212.3 ± 4.5^a^	54.9 ± 1.9^a^, ^b^

Note

Dara are expressed as the mean ± SEM.

^a^ *P* < .05 compared with db/m + vehicle group.

^b^ *P* < .05 compared with db/db + vehicle group.

### 3.2 SAL ameliorated diabetes‐induced glomerular injury in db/db mice

**FIGURE 1:**
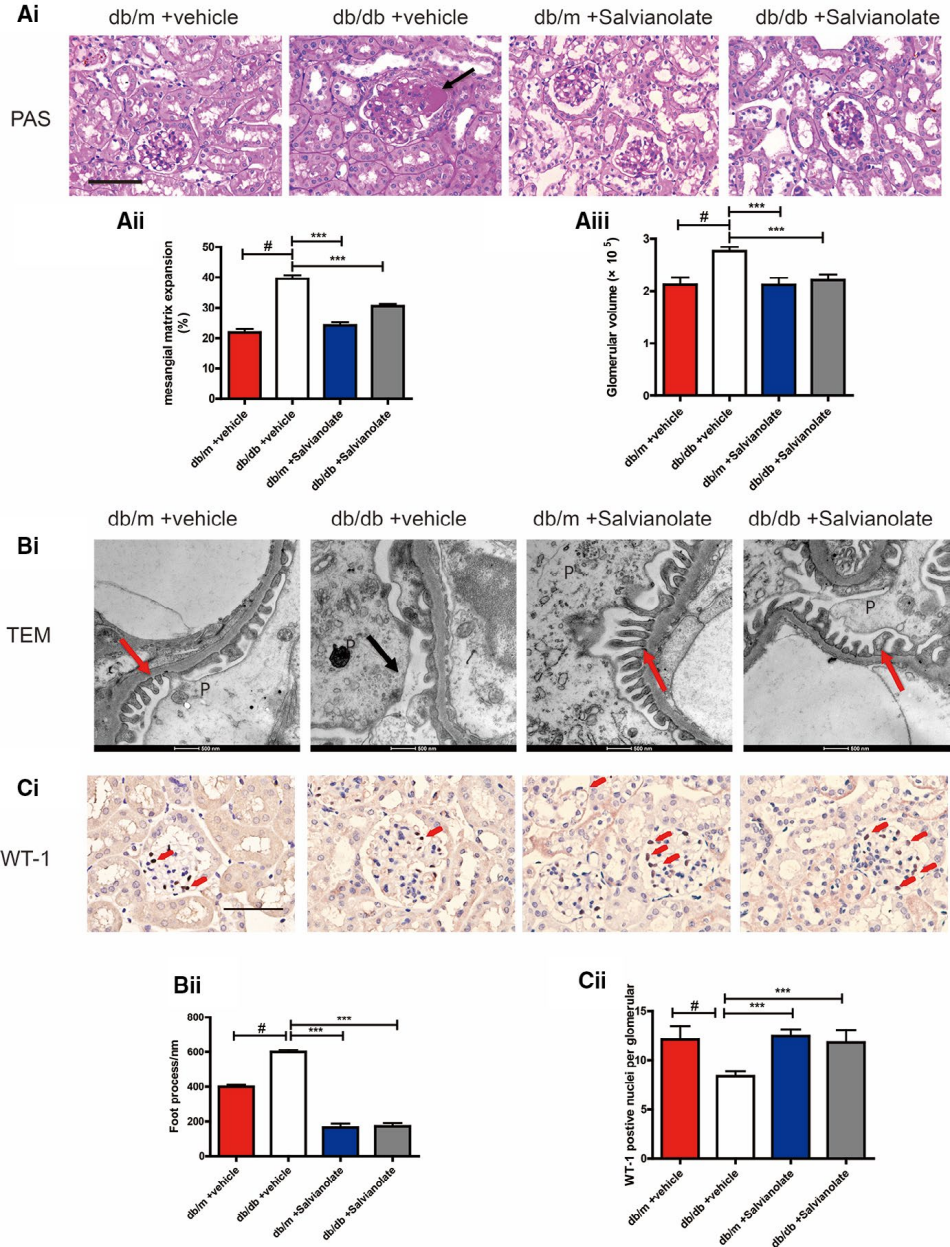
Effects of SAL on diabetic nephropathy related histological features. A(i), representative images of periodic acid‐schiff (PAS)‐stained kidney sections (original magnification × 200, Bar = 50 μM). A(ii), quantification analysis of mesangial matrix expansion. A(iii), quantification of glomerular volume analysis of different groups. B(i), Representative TEM images of podocytes (P). black arrows indicated areas of foot process (FP) effacement. Red arrows indicated a normal foot process. B(ii), quantification analysis of foot process width. C(i), immunochemistry staining of representative kidney sections for WT‐1 in glomeruli (original magnification × 400, Bar = 100 μM, the red arrow indicates the positive nuclei). C(ii), quantification analysis of the average number of WT‐1‐positive cells per glomerular respectively. ^#^ *P* < .001 compared with vehicle‐treated db/m group (n = 6). ^**^ *P* < .01, ^***^ *P* < .001 vs vehicle‐treated db/db mice (n = 6)

One of the pathological features of DN is severe mesangial hyperplasia and podocytes loss. Periodic Acid‐Schiff staining revealed that SAL alleviated mesangial matrix deposition in glomerulus compared with control db/m mice (Figure 1A). As mentioned above, this was consistent with the mitigating effect of SAL on albuminuria of db/db mice. Transmission electron microscopy of kidney sections showed SAL treatment significantly alleviated severe and diffuse effacement of foot process in the glomeruli of db/db mice compare with saline treated db/db mice (Figure 1B). Furthermore, we found that db/db mice treated with NOX1/4 inhibitor GKT137831 for 16 weeks resulted in similar renoprotection as observed in SAL treated db/db mice, as assessed by alleviated mesangial hyperplasia, podocyte foot process effacement in glomeruli (Figure S2).

### 3.3 SAL prevented podocyte loss in db/db mice

**FIGURE 2:**
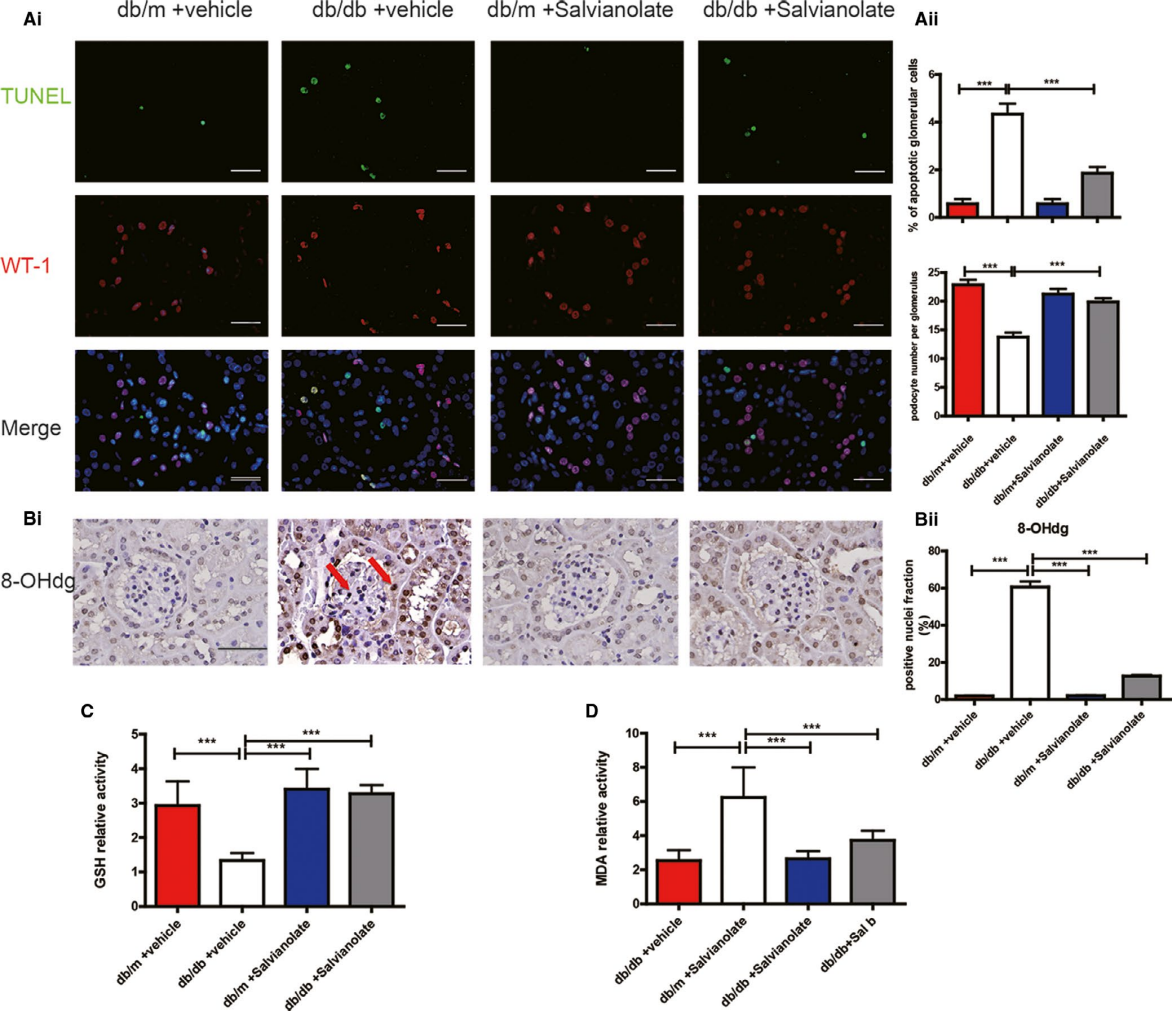
SAL attenuated renal oxidative stress in db/db mice. A(i), Kidney sections also were used for co‐staining transferase dUTP nick end labelling (TUNEL) and wild‐type 1 (WT‐1) to determine the rate of apoptosis in podocytes. A(ii), Quantification of TUNEL‐positive cells per glomerulus and the number of podocytes per glomerulus. ^**^ *P* < .01, ^***^ *P* < .001 vs vehicle‐treated db/db mice (n = 6). B(i), representative kidney sections immunostained for 8‐OHdG to show oxidative stress of kidney tissue (original magnification × 400, Bar = 100 μM, the red arrow indicates the positive nuclei). B(ii), quantification of 8‐OHdG‐positive cells fraction. C, Effects of SAL on GSH level in renal cortex from different intervened group. D, Effects of SAL on malondialdehyde (MDA) activity in renal cortex from different intervened groups; ^***^ *P* < .001 vs vehicle‐treated db/db mice (n = 6)

To determine the role of SAL on podocyte loss, we performed immunostaining for podocyte marker WT‐1 in diabetic kidney sections. SAL treatment increased the number of WT‐1‐positive cells per glomerulus of db/db mice (Figure 1C). The expression of differentiated podocytes markers synaptopodin showed similar results (Figure S3). We evaluated apoptosis podocyte in db/db mice by Co‐labelling WT‐1 and TUNEL. TUNEL analysis revealed significantly increased apoptotic podocyte in the glomeruli of saline treated db/db mice, which can be reversed by SAL treatment (Figure 2A). These findings suggested that SAL treatment attenuated podocyte injury in db/db mice.

### 3.4 SAL attenuated renal oxidative stress in db/db mice

Considering that renal injury in diabetes is associated with increased formation of ROS, we measured 8‐hydroxy‐2 deoxyguanosine (8‐OHdG) levels, a marker of oxidative DNA damage, in renal cortex of diabetic mice. The expression intensity of 8‐OHdG was significantly decreased in SAL treated db/db mice compared with saline treated db/db mice (Figure 2B). Moreover, SAL treatment rescued the decreasing level of the antioxidant glutathione (GSH) of db/db mice (Figure 2C). Oxidative stress‐related enzymes and renal malondialdehyde (MDA) level in SAL treated db/db mice was also reduced compared with db/db control mice, suggesting a lower lipid peroxidation level in renal tissue (Figure 2D). Briefly, SAL treatment alleviated hyperglycaemia induced renal oxidative stress in db/db mice.

### 3.5 SAL suppressed NOX4‐based NADPH oxidase activity in db/db mice

**FIGURE 3:**
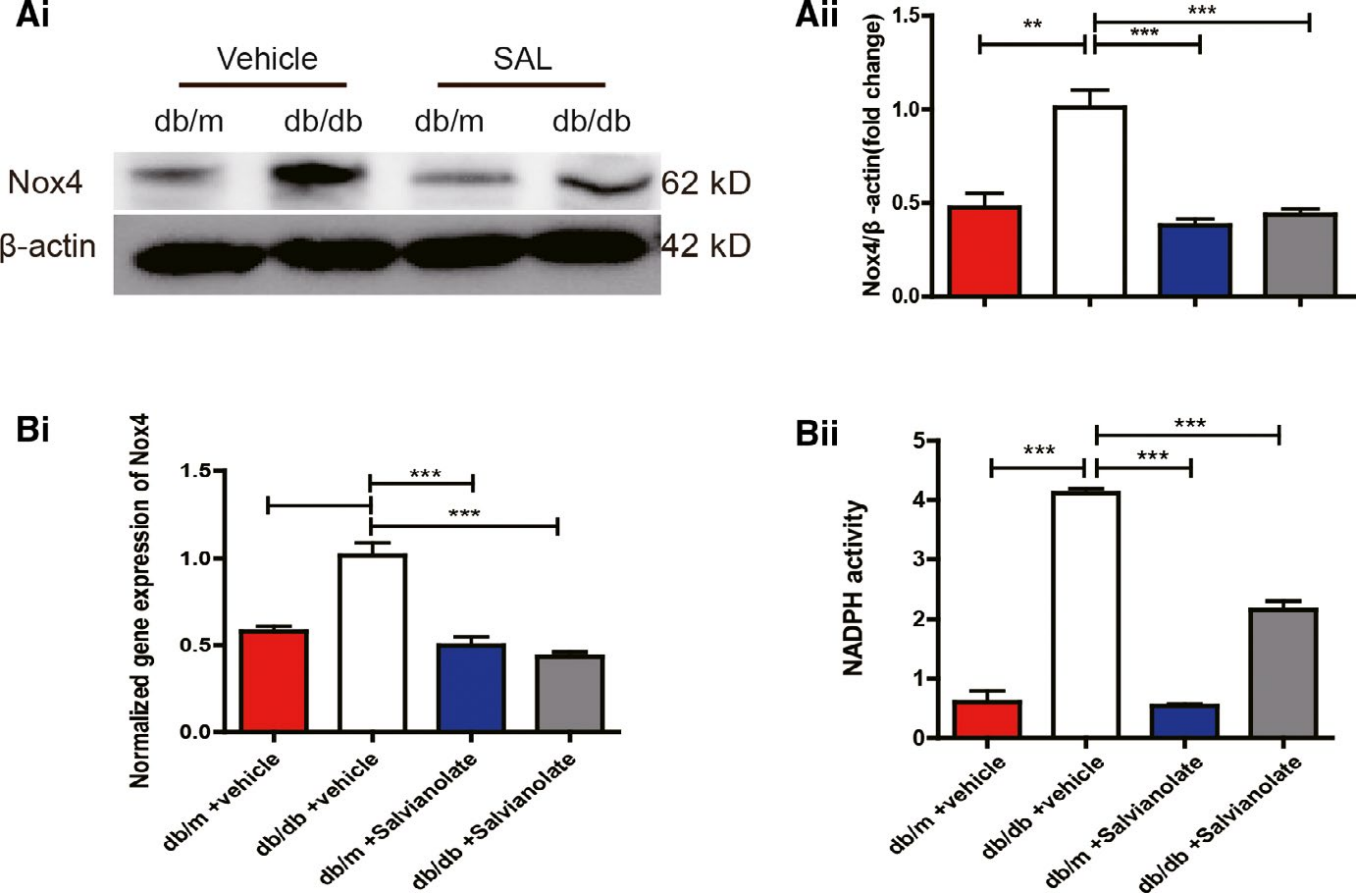
Treatment of db/db mice with SAL decreased NOX4 expression and NADPH oxidase activity. A(i), representative immunoblots of NOX4 expression of glomeruli. A(ii), quantitative results of immunoblots from A(i). B(i), glomeruli mRNA levels of NOX4 from different intervened groups were assessed by real‐time PCR. B(ii), histogram representing NADPH oxidase activity in glomeruli lysates from different intervened groups. ^**^ *P* < .01, ^***^ *P* < .001 vs vehicle‐treated db/db mice (n = 6)

The effect of SAL on NOX4 was reflected in the three levels of transcription, translation and enzyme activity. Considering that NOX4 was the main source of oxidative stress in the kidney of DN, we explored the effect of SAL on the NOX4 expression and activity. An increase in expression of NOX4 in glomerular of db/db control mice was markedly attenuated after treatment with SAL (Figure 3A). These effects were paralleled by changes in the mRNA level of NOX4 (Figure 3B(i)). We also measured superoxide production by the NADPH oxidase in the glomeruli using the chemiluminescence of lucigenin. Activation of NAPDH oxidase in the glomeruli was found in db/db mice, whereas SAL decreased the NADP+/NADPH ratio of db/db mice (catalysed by NADPH oxidase NOX4) (Figure 3B(ii)). These findings supported that SAL suppressed the expression of NOX4 at the gene and protein levels and finally enzyme activity from all sides.

### 3.6 Sal B blocked HG‐induced NOX4‐based ROS generation and apoptosis in human podocytes

It was previously found that oxidant injury to podocytes may play a pivotal role in the pathogenesis of DN.^26^ Currently, Sal B is used as a quality‐control ingredient and active marker for Danshen products by the National Pharmacopoeia Council of China. Based on this, Sal B was used to test the in vitro efficacy of SAL on the high glucose‐induced podocyte oxidant injury in a human differentiated podocyte cell line as previously described.^27^

**FIGURE 4:**
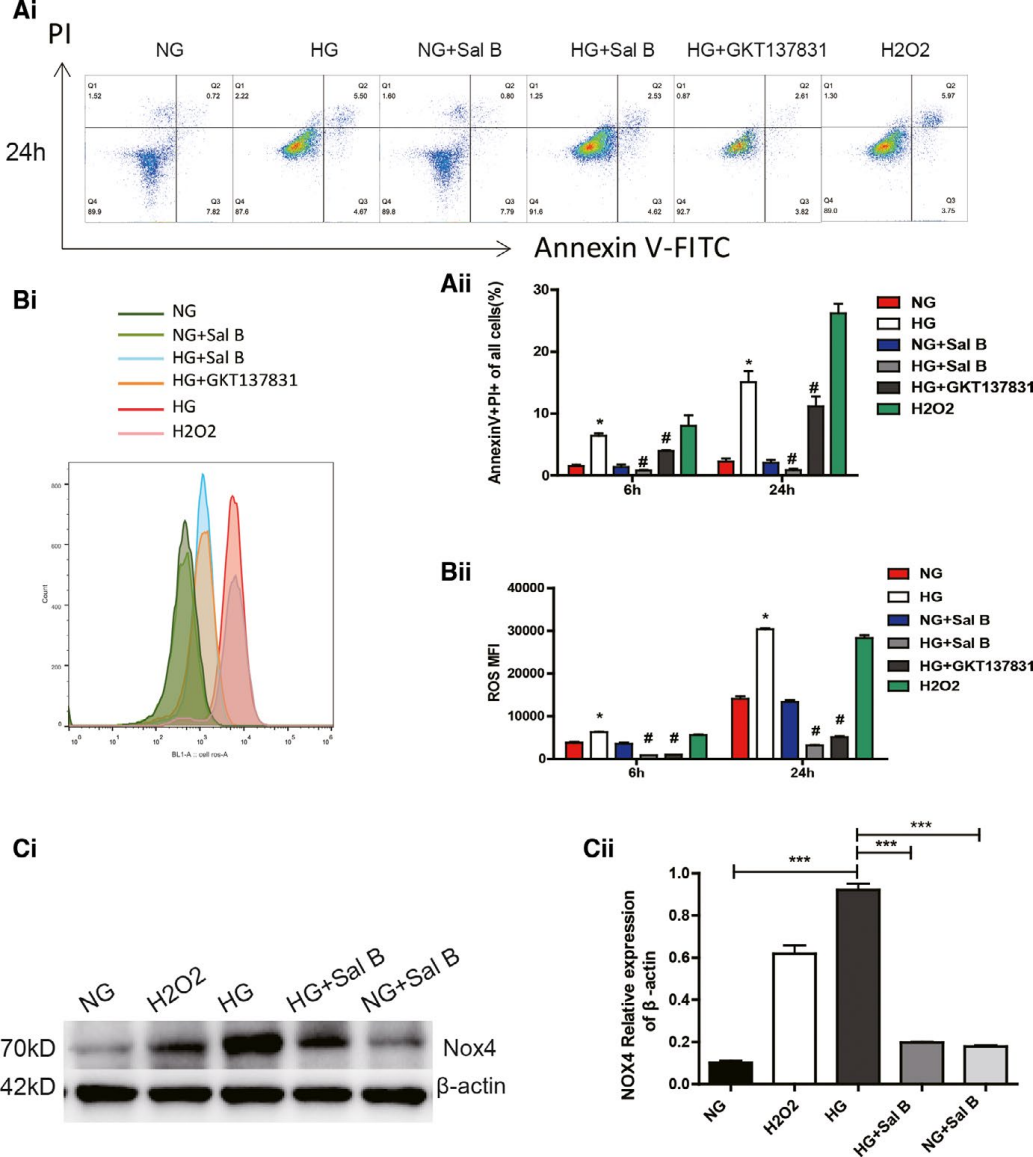
Effects of Sal B on HG‐induced podocytes ROS generation and apoptosis. Human podocytes were exposed either to HG (35 mM) and with or without the Sal B (200 µM), GKT137831 (20 µM) or to H_2_O_2_ (200 µM) in NG(5 mM) for 24 h: A(i), podocytes apoptosis examined by flow cytometry assay A(ii), quantification of per cent annexin V‐positive cells. B(i), ROS measurement using 2,7‐dichlorofluorescein (DCF). B(ii), statistical analysis of ROS fluorescence intensity (MFI) values. C(i), representative western blot of Nox4 levels in podocytes. C(ii), western blots from three experiments were quantified by densitometry analysis. Values are presented as the means ± SEM (^*^ *P* < .05, ^***^ *P* < .001 vs NG group; ^#^ *P* < .05 vs HG group)

As shown in Figure 4A,B, exposure of human podocytes to 35 mmol/L high glucose (HG) for 24h resulted in a significant increase in the apoptosis rate and intracellular H_2_O_2_ level as assessed by Annexin V‐FITC and DCFH‐DA fluorescence respectively. H_2_O_2_ (200 uM) incubation was used as positive control of NOX4 activation, while NOX1/NOX4 inhibitor GKT137831 (20 uM) incubation was used as a negative control to NOX4 expression. The high glucose induced increase in ROS production as well as apoptosis rate were reduced in podocytes treated with Sal B or GKT137831 (20 µM; Figure 4A,B). Besides, Sal B treatment also ‐markedly decreased the HG‐induced increase in Nox4 protein level (Figure 4C). These results were consistent with in vivo relevance of the findings in db/db diabetic mice.

### 3.7 AMPK is required for Sal B to attenuate NOX4‐induced podocyte apoptosis

**FIGURE 5:**
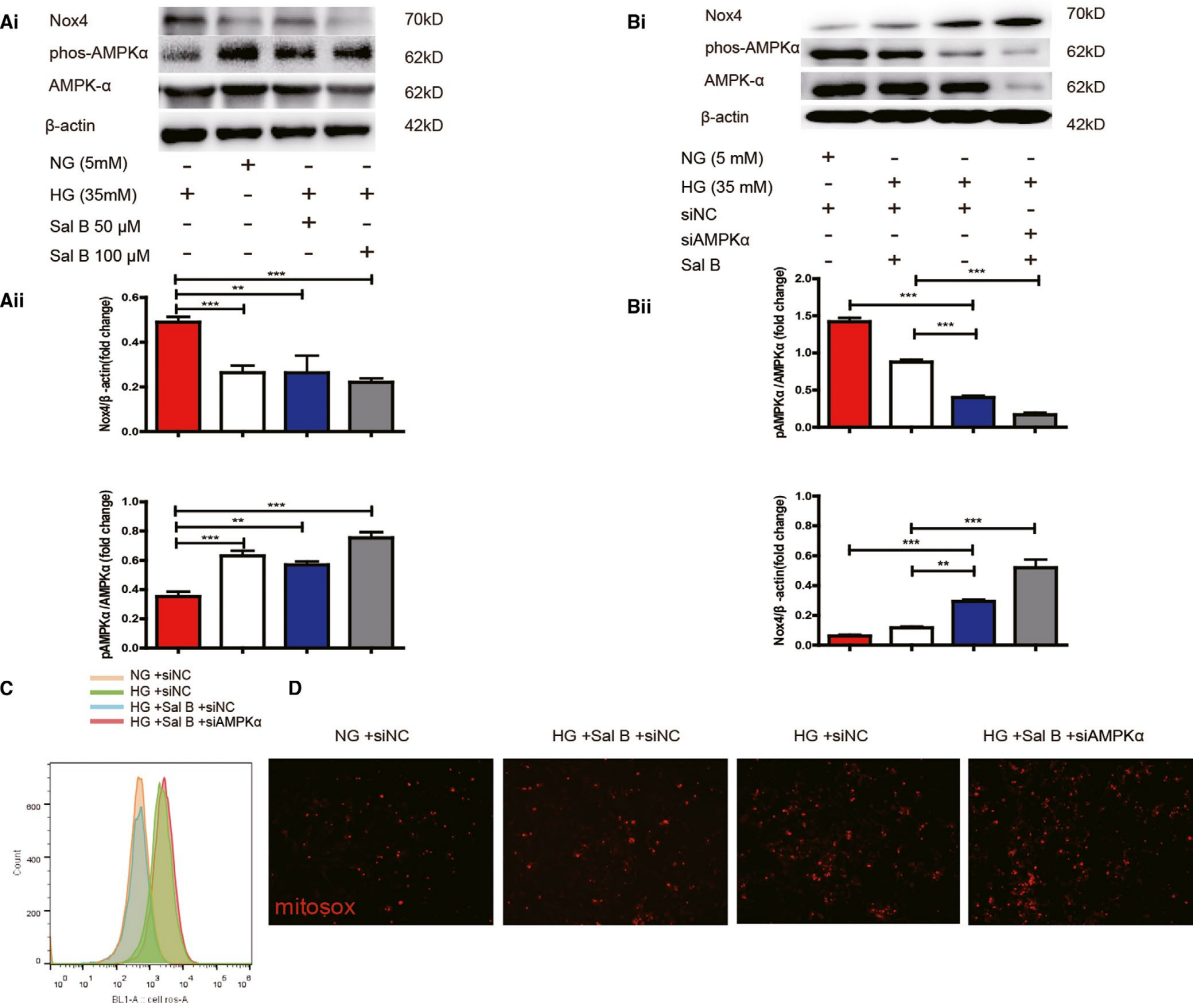
Sal B alleviated HG‐induced NOX4 up‐regulation part through AMPK regulation. A(i), representative western blot of p‐AMPKα^Thr172A^,AMPKα, NOX4. A(ii), histogram representing AMPK activity measured in podocytes treated in NG (5 mM) or HG (35 mM) and in the presence or absence of Sal B (50 µM, 200 µM) for 24 h. B(i), In parallel experiments, podocytes were transfected with siNC in NG or transfected with siRNA‐AMPKα in HG and in the presence or absence of Sal B (200 µM) for 24 h. Shown is a representative western blot of p‐AMPKα^Thr172A^, AMPKα, NOX4.B(ii): histogram representing AMPK and NOX4 activity measured as indicated. C, ROS measurement using 2,7‐dichlorofluorescein (DCF) D, MitoSOX (red) immunofluorescence in podocytes cultured as described in B. The results were from three independent experiments expressed as the means ± SEM (^*^ *P* < .01, ^**^ *P* < .01, ^***^ *P* < .001 vs NG group)

Previous studies had brought forward AMPK to be an upstream regulator of NOX4.^28^, ^29^ To determine whether Sal B exerted anti‐oxidative effects via AMPK regulation, AMPK expression was knocked down by siRNA. Significant reduction in AMPK protein expression (40%) was observed 24 hours after siRNA transfection. First, in cells incubated with HG, treatment with Sal B restored AMPK phosphorylation at Thr172 as well as AMPK activity. Sal B also prevented the increased in NOX4 protein expression in response to HG (Figure 5A). Secondly, inhibition of AMPK with siRNA‐AMPKα robustly enhanced the basal expression of Nox4 protein in HG‐induced podocytes, while siRNA‐AMPKα also abrogated the effect of Sal B on NOX4 inhibition (Figure 5B). Thirdly, to confirm that mitochondrial NOX4‐based ROS is responsible for glucose‐induced podocyte injury, a combination of NOX4 and Mitotracker green staining was performed in podocytes incubated with HG for 24 hours. Immunostaining of Nox4 using rabbit monoclonal antibody clearly colocalized with Mitotracker, as indicated by the presence of multiple yellow puncta in the merged image (Figure S4), implying that Nox4 was primarily localized to mitochondria in podocytes. In addition, transfection with siRNA‐AMPK blocked the protective effect of Sal B against HG‐induced ROS (Figure 5C) and mitochondria‐derived ROS generation (Figure 5D) as evidenced by Flow cytometry and MitoSOX™ staining respectively. These data strongly suggest that Sal B possesses the reno‐protective capabilities in part through AMPK‐mediated control of NOX4 expression.

### 3.8 Sal B inhibited HG‐induced podocyte apoptosis via blockade of mitochondrial NOX4

**FIGURE 6:**
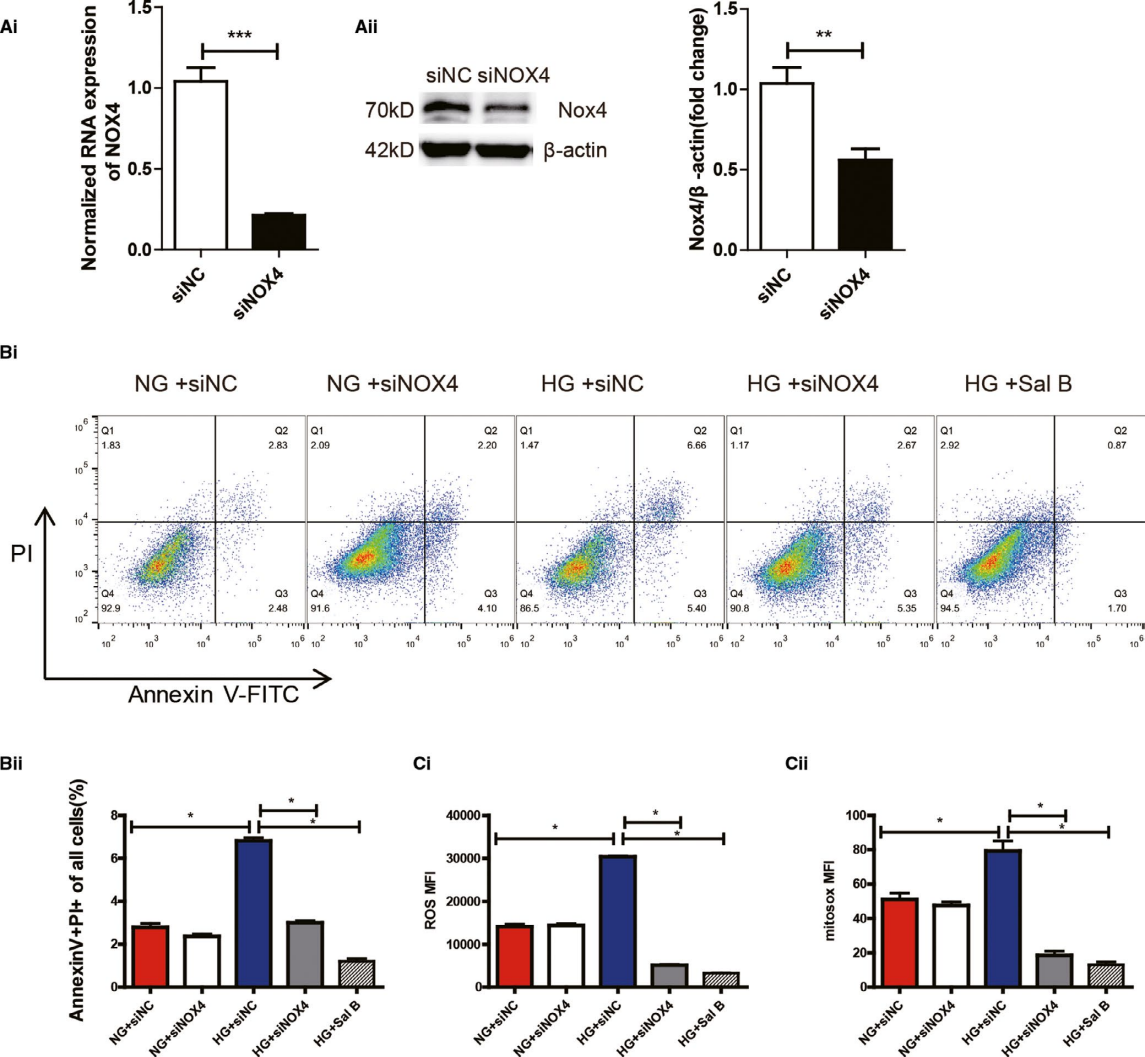
Effect of Sal B on oxidative stress mediated podocyte apoptosis is associated with down‐regulation of NOX4. A(i), Effect of Nox4 siRNA and siNC (24 h) on Nox4 protein expression in podocytes. A(ii), Relative mRNA amount of NOX4. B(i), Apoptosis of podocytes intervened by siNOX4 detected by flow cytometry using Annexin V‐PI staining B(ii), Quantification of per cent annexin V‐positive cells. C(i), Histogram representing ROS fluorescence intensity (MFI)values in podocytes transfected with siNC or siRNA‐NOX4 in HG or NG and in the presence or absence of Sal B (200 uM) for 24h. C(ii), Histogram representing MitoSOX fluorescence intensity (MFI) values in podocytes cultured as described in C(i). The results were from three independent experiments expressed as the means ± SEM (*P < .05; **P < .01; ***P < .001 vs NG group)

To further elucidate the exact mechanism of Sal B on NOX4 regulation, we next infected human podocytes with small interfering RNAs (siRNA) expression construct. Effective down‐regulation of Nox4 protein by siRNA in human podocytes was confirmed by the results shown Figure 6A. Impairment of Nox4 function with siRNA and Sal B treatment resulted in the similar reduction in HG‐induced increase in ROS generation and TUNEL‐positive nuclei in human podocytes (Figure 6B,C).Moreover, as detected by MitoSOX^TM^ staining, Sal B treatment caused significant reduction in the increased mitochondria‐derived ROS production mediated by HG, similar to the effects of siRNA‐NOX4 (Figure 6C). In addition, silencing of NOX4 did not abrogate HG dependent increase in oxidative stress and apoptosis in podocytes, suggesting that Sal B may ameliorate glucose‐induced podocyte injury part through impeding mitochondrial NOX4 up‐regulation by HG.

## 4 DISCUSSION

SAL is the aqueous bioactive component extracted from the Chinese herbal medicine Salvia miltiorrhiza (Danshen), which has been widely and successfully used for microvascular diseases. Sal B is the representative active marker and quality‐control ingredient of SAL.^30^ In our study, we found SAL attenuated podocytes loss, foot process effacement, proteinuria and hyperglycaemia (without significant difference) in diabetic mice. What's more, we also confirmed Sal B inhibited high glucose‐induced NOX4‐derived ROS overproduction by regulating AMPK activation (Figure S5). These findings provided a deeper insight into the medical application of Sal B in treating DN.

This is the first study to delineate the effect of Sal B on the diabetic db/db mice, focusing on glucose‐induced oxidative damage and podocyte injury. Previous studies from our group and others have since confirmed the correlation between podocyte loss, proteinuria and glomerulosclerosis, indicating that podocyte injury played a pivotal role in the pathogenesis of DN.^22^, ^27^, ^31^ In the current study, we observed that SAL not only rescued the loss of podocytes in diabetic mice, but also attenuated the foot process effacement of podocytes which was part of an important barrier of GBM. These protective effects may be associated with the remission of proteinuria in diabetic mice.

Sal B has been proven to provide antidiabetic effect, which may relate to several mechanisms such as anti‐oxidative, insulin sensitizing, anti‐inflammatory properties.^9^, ^14^ Accumulating evidence demonstrated that the glucose‐induced oxidative stress emerged as a critical pathogenic factor in DN.^4^ In addition, Sal B has been proved to be among the most effective natural antioxidants due to its polyphenolic structure.^32^ Our results showed that SAL alleviated the oxidative stress in the diabetic kidneys, which was reflected by a reduction in 8‐OHdG, a marker of oxidative DNA damage as well as MDA lipid peroxidation, paralleling with augmentation of antioxidant GSH. These data are consistent with our previous findings that SAL possessed the reno‐protective capabilities via scavenging ROS.^19^

The association between NOX‐mediated oxidative damage and the pathogenesis of DN has been well studied.^33^ In the rodent kidney, four isoforms of the catalytic subunit of NADPH oxidase are expressed (NOX1, NOX2, NOX4 and NOX5).^6^ Particularly the isoform NOX4 is a critical mediator of redox signalling in podocytes exposed to the diabetic milieu.^34^ With regard to in vivo studies, genetic deletion of NOX4 globally and specifically in podocytes or administration of a novel NOX1/4 inhibitor (GKT137831) attenuated the development of DN.^34^, ^35^, ^36^ Consistent with this view, we observed augmented NADPH oxidase activity and increased expression of NOX4 in the cultured human podocyte exposed to HG, as well as in vivo in glomeruli of diabetic mice. Indeed, we demonstrated both SAL and NOX inhibitor (GKT137831) treatment caused similar reduction in increase of NOX4 based ROS generation and apoptosis in diabetic kidney and HG‐induced podocytes. Mitochondrial ROS has been shown to be the major player that contributes to progression of DN.^37^, ^38^ In current study, we found that NOX4 was expressed in the mitochondrial compartment and Sal B treatment blocked glucose‐induced mitochondrial superoxide generation as detected by MitoSOX, suggesting that Sal B could prevent glucose‐induced oxidative stress through impeding mitochondrial NOX4 up‐regulation by HG in podocytes. In addition, silencing of NOX4 using siRNA approach did not abrogate the effect, suggesting that Sal B may ameliorate podocyte injury part through blockade of NOX4‐dependant ROS signalling in DN.

It has been well documented that AMPK might function as a negative modulator of NOX activation and thus protect against oxidative stress in diabetes.^39^, ^40^, ^41^ It was recently described that high glucose‐induced NOX4 up‐regulation and NOX4 mediated podocyte apoptosis could be decreased by AMPK activation in human podocytes.^42^ Consistent with this view, we showed that Sal B prevented HG‐induced AMPK inactivation in podocytes. This is in agreement with the data from the literature showing that AMPK phosphorylation was reduced by nearly 70% in renal cortex of db/db mice, a model of T2DM.^17^ Our results also indicated that Sal B administration could inhibit the induction of NOX4 and NADPH activity by HG and prevent the subsequent enhanced ROS production, thereby attenuating podocyte injury, which can be abrogated by AMPK knockdown. These results showed Sal B exerted its antioxidant activity against NOX4‐derived oxidative stress via AMPK activation. Future studies examining podocyte‐specific NOX4‐deficient mice is required to confirm the involvement of the AMPK/NOX4 pathway in the protective role of Sal B against podocyte injury in DN.

Furthermore, our results showed that compared with saline treated db/db mice, there is a slight reduction of blood glucose in db/db mice receiving sixteen‐week treatment, of SAL whereas the difference in glucose lowering between SAL and saline treated db/m control mice was unchanged throughout. Raoufi, S demonstrated that three‐week SAL (20 and 40 mg/kg) treatment caused a significant decrease of the serum glucose by protecting pancreatic beta cell against oxidative stress and apoptosis in STZ induced diabetic rats^9^; Huang, M also found that six‐week treatment of diabetic rats with SAL (100 and 200 mg/kg) significantly decreased blood glucose through improving insulin resistance(IR) and antioxidant activity^14^; However, the different results from these studies may be explained by the experimental conditions, such as duration of the experimental model, cell types, dose and timing of treating. Moreover, previous study showed that the hypoglycaemic mechanisms of many polyphenolic compounds in medical plants such as green tea catechins, pomegranate polyphenols are closely related to their antioxidant activities. With this knowledge combined with our observations, we propose that Sal B has the potential to effectively prevent DN due to its antioxidant properties.

Finally, at present, mitochondrial dysfunction appears to be a key event leading to DN, and specific targeting of mitochondrial ROS may yet prove to be beneficial in diabetes.^43^ Consistent with this concept, our findings showed that Sal B prevents podocyte injury via targeting of mitochondrial NOX4 mediated ROS generation under hyperglycaemia conditions. In addition, it has been shown that hyperglycaemia mediated mitochondrial oxidative damage of glomerular endothelial cells plays an essential role in mediating podocyte injury and subsequent progression of DN.^44^, ^45^ However, we need more evidence such as endothelial cell selective NOX4 knockout mice or rationally designed clinical trial of SAL in patients with DN to explore the mechanisms underlying the reno‐protective role of Sal B in DN.

## 5 CONCLUSIONS

Diabetic nephropathy (DN) is the leading cause of ESRD as it affects as much as one‐third of diabetics. Our data firstly provided the evidence showing that Salvianolate treatment attenuated diabetes‐induced glomerular injury, including albuminuria secretion, mesangial matrix expansion, foot process effacement and ECM accumulation in the kidneys of db/db mice. We also provide new insights concerning the molecular mechanism involved in these events and demonstrate that Salvianolate ameliorated oxidative podocyte injury via modulation of NOX4 activity. We also raise the possibility that Sal B could be a promising natural compound to rescue the podocyte from oxidative damage and prevent subsequent progression of DN. There is still a long way to bring the medical application of Sal B on the clinical therapy of DN.

## CONFLICT OF INTEREST

The author declares that there is no conflict of interest that could be perceived as prejudicing the impartiality of the research reported .

## AUTHOR CONTRIBUTION

**Yiran Liang:** Investigation (lead); Methodology (lead); Writing‐original draft (equal). **Hong Liu:** Conceptualization (equal); Supervision (equal); Visualization (equal). **Yi Fang:** Methodology (supporting); Resources (equal). **Pan Lin:** Methodology (equal); Software (equal). **Zhihui Lu:** Project administration (equal); Software (equal). **Pan Zhang:** Conceptualization (equal); Resources (equal). **Xiaoyan Jiao:** Investigation (equal); Validation (equal). **Jie Teng:** Conceptualization (equal); Resources (equal). **Xiaoqiang Ding:** Funding acquisition (lead); Resources (lead). **Yan Dai:** Investigation (supporting); Supervision (supporting); Writing‐original draft (lead); Writing‐review & editing (lead).

## ETHICAL APPROVAL

All animal studies were performed according to the protocols approved by the Institutional Animal Care and Use Committee of Fudan University and conducted following the National Institutes of Health Guide for the Care and Use of Laboratory Animals.

## Supporting information

Fig S1

Fig S2

Fig S3

Fig S4

Fig S5

Table S1
