# Supplementary figures and images for: Salvianolate ameliorates oxidative stress and podocyte injury through modulation of NOX4 activity in db/db mice
Source: J Cell Mol Med. 2020 Dec 17;25(2):1012–23. doi: 10.1111/jcmm.16165 (PMC7812253; doi:10.1111/jcmm.16165)

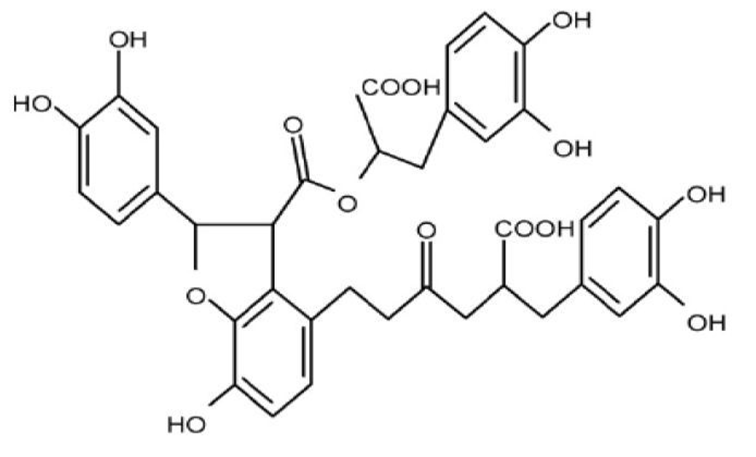

Supplement: Supplementary file 1 — Fig S1 [file JCMM-25-1012-s001.tif]

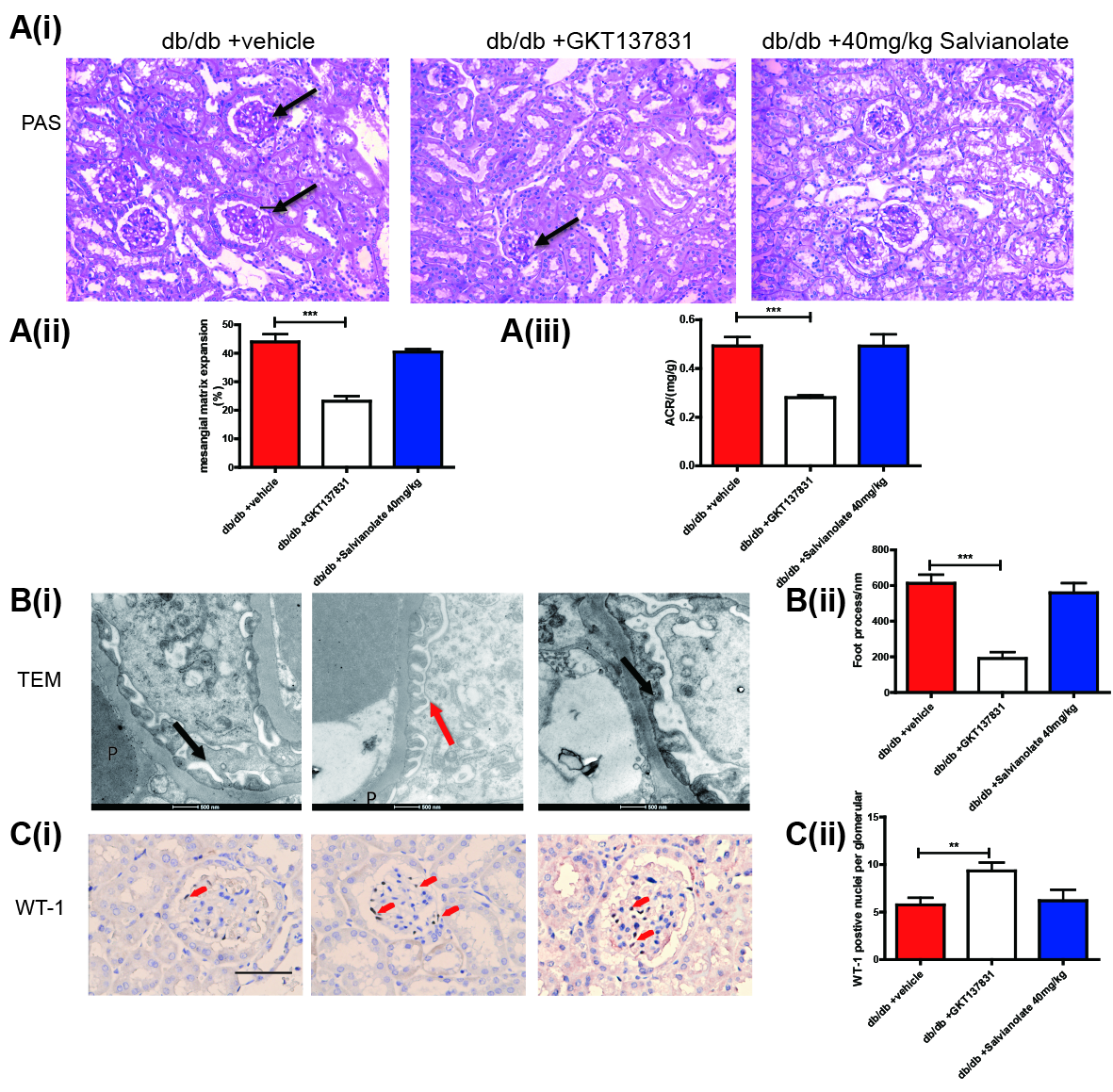

Supplement: Supplementary file 2 — Fig S2 [file JCMM-25-1012-s002.tif]

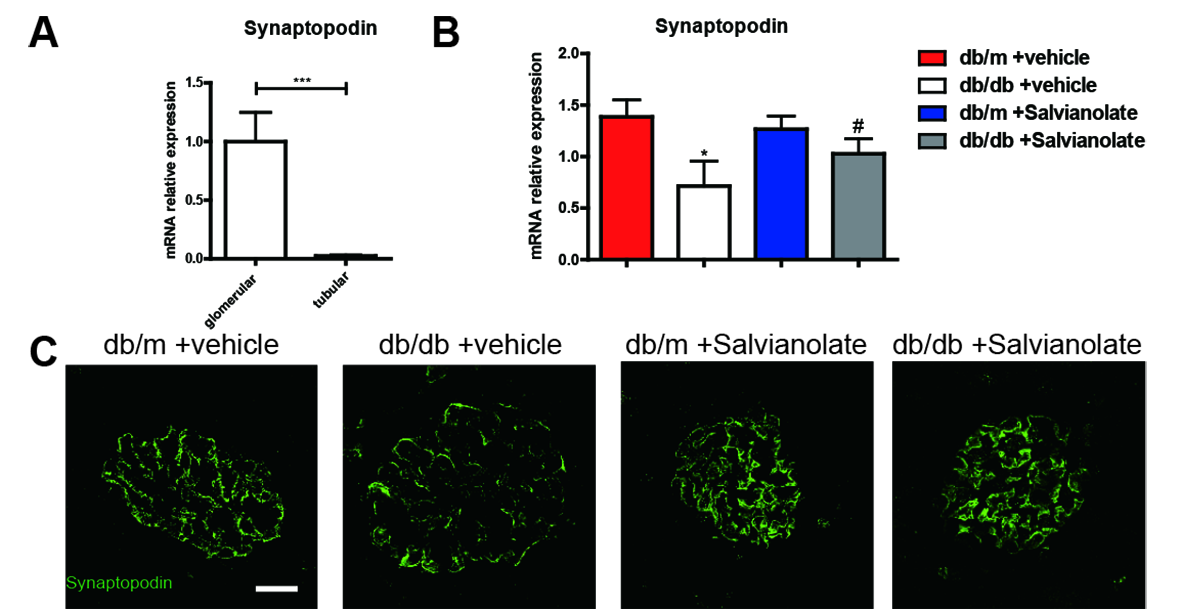

Supplement: Supplementary file 3 — Fig S3 [file JCMM-25-1012-s003.tif]

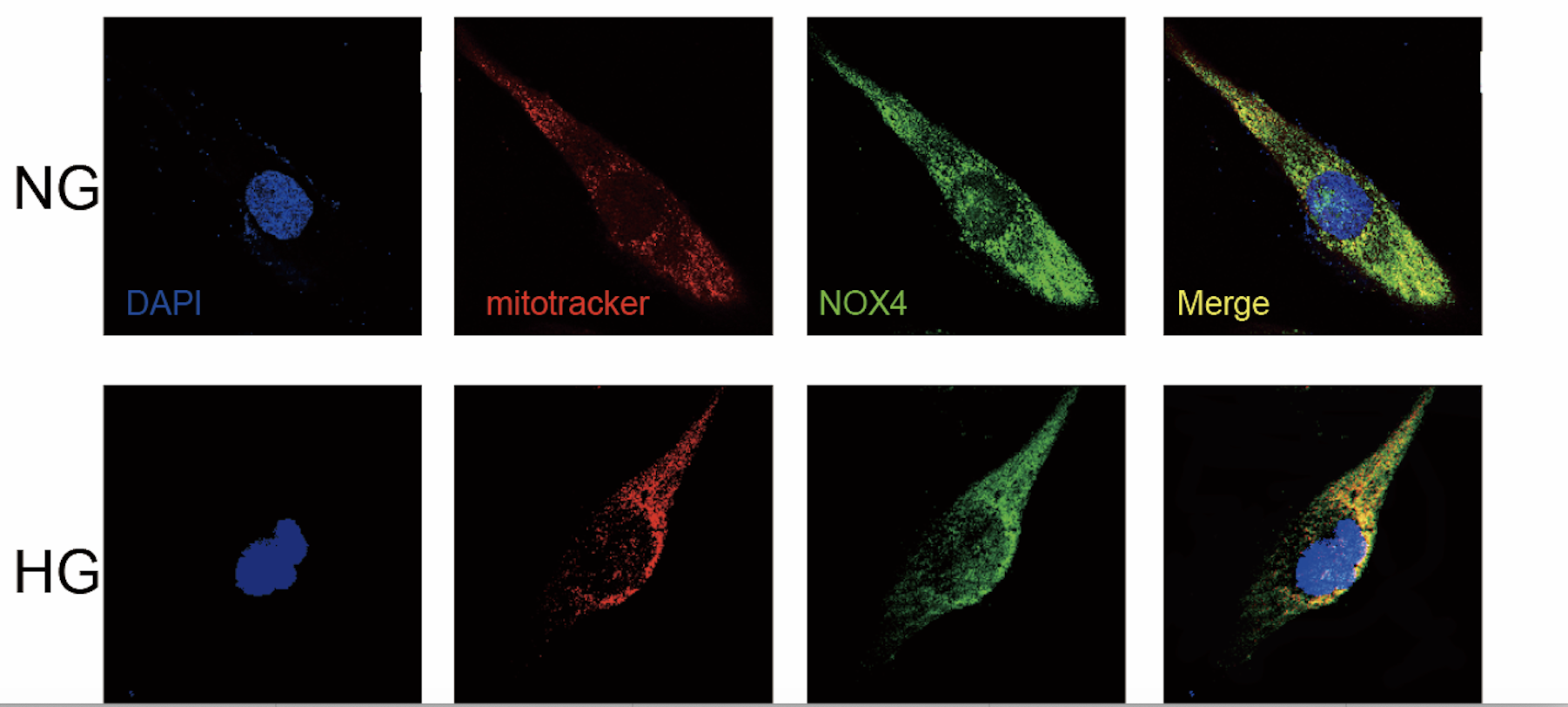

Supplement: Supplementary file 4 — Fig S4 [file JCMM-25-1012-s004.png]

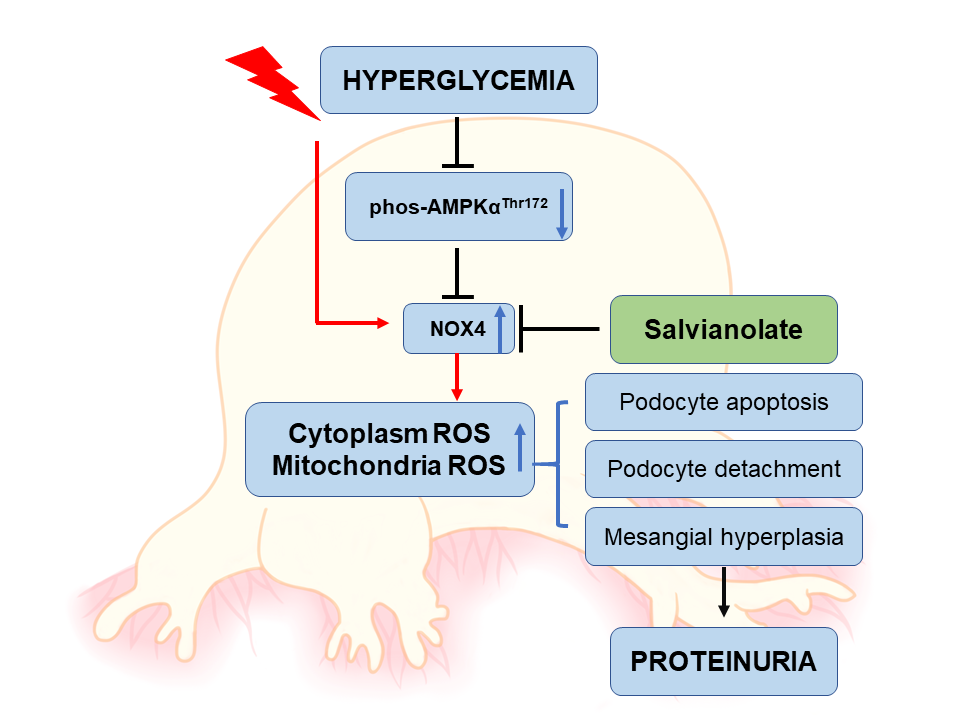

Supplement: Supplementary file 5 — Fig S5 [file JCMM-25-1012-s005.tif]
