# Supplementary material for: Salvianolate ameliorates oxidative stress and podocyte injury through modulation of NOX4 activity in db/db mice
Source: J Cell Mol Med. 2020 Dec 17;25(2):1012–23. doi: 10.1111/jcmm.16165 (PMC7812253; doi:10.1111/jcmm.16165)
Supplement: Supplementary file 6 — Table S1 [file JCMM-25-1012-s006.docx]

**Supplementary Table 1. Real-time PCR primers**

| Gene | Forward Primer | Reverse Primer |
| --- | --- | --- |
| β-actin | 5’-AGCCATGTACGTAGCCATCC-3’ | 5’-GCTGTGGTGGTGAAGCTGTA-3’ |
| NOX4 | 5’-CTTCACAACTGTTCCTGGCC-3’ | 5’-TTCTGAGAGCTGGTTCGGTT-3’ |
| Synaptopodin | 5’-CTTTGGGGAAGAGGCCGATTG-3’ | 5’-GTTTTCGGTGAAGCTTGTGC-3’ |
